# “Crack babies” : the management protocol

**DOI:** 10.1186/1824-7288-40-S2-A42

**Published:** 2014-10-09

**Authors:** Fedora del Prato, Romilda Ferraro, Concetta Pellecchia, Maria Sellitto

**Affiliations:** 1 Neonatal Intensive Care Unit, Villa Betania Evangelical Hospital, Naples, Italy

## Introduction

During the pregnancy maternal drugs use represent an important socio medical issue for their children. In the USA, 10% of women takes drugs during pregnancy: 1-2% use heroin, 3-4% cocaine and less than 1% cannabinoids [1]. The phenomenon is increasing and the country and the neonatal divisions need new management protocols. In Italy children of a mother drug addict born as late preterm are the 17- 29%. The “crack babies” are children exposed to drugs during the pregnancy: we can already define them drug addicts. They may show intrauterine (choking, infection, malformations), neonatal (withdrawals symptoms, prematurity, respiratory distress syndrome, growth restriction neonatal hyperbilirubinemia), and postnatal complications (delay in psychomotor, deficit in language, SIDS, learning disabilities, difficulties in concentration, instability etc)[2] .

In some countries several projects about essential care have been elaborated and realized. In Campania people involved in this field are pediatricians, child psychiatrists, psychologists, teachers judges, social assistants, without a real defined cooperation.

## Network

Protocol provides hospital stay of drug addict woman during the labour to guarantee assistance and monitoring of the mother and the child [3]. For this reason it has been created a sociology service to coordinate the network. System facilities are: the Ser.T (services for drug addicts) to help the mother, a territorial social office which provides to the child, the Minor’s court, the hospital for the psychological and physiological children development (if he was born with Newborn Abstinence Syndrome) and the obstetrical service.

## Conclusion

Our protocol has two main targets:

• Safe the health, the security and the wellness of the child even after the discharge trough periodical check-up of follow-up (DH);

• Facilitate the development of relationship between mother and child to reduce the necessity of Minor’s court interventions.

Only in this way it will be possible to realize a real and integrated takeover of the mother-child entity.
